# Integrated FISH, Karyotyping and aCGH Analyses for Effective Prenatal Diagnosis of Common Aneuploidies and Other Cytogenomic Abnormalities

**DOI:** 10.3390/medsci7020016

**Published:** 2019-01-23

**Authors:** Hongyan Chai, Autumn DiAdamo, Brittany Grommisch, Jennifer Boyle, Katherine Amato, Dongmei Wang, Jiadi Wen, Peining Li

**Affiliations:** Laboratory of Clinical Cytogenetics, Department of Genetics, Yale School of Medicine, New Haven, CT 06520, USA; hongyan.chai@yale.edu (H.C.); autumn.diadamo@yale.edu (A.D.); brittany.grommisch@yale.edu (B.G.); jennifer.boyle@yale.edu (J.B.); Katherine.wilcox@yale.edu (K.A.); dongmei.wang@yale.edu (D.W.); jiadi.wen@yale.edu (J.W.)

**Keywords:** fluorescence in situ hybridization (FISH), karyotype, array comparative genomic hybridization (aCGH), amniotic fluid (AF), chorionic villus sampling (CVS), aneuploidies, pathogenic copy number variants (pCNV), confined placental mosaicism (CPM), true fetal mosaicism (TFM), pseudo-mosaicism

## Abstract

Current prenatal genetic evaluation showed a significantly increase in non-invasive screening and the reduction of invasive diagnostic procedures. To evaluate the diagnostic efficacy on detecting common aneuploidies, structural chromosomal rearrangements, and pathogenic copy number variants (pCNV), we performed a retrospective analysis on a case series initially analyzed by aneuvysion fluorescence in situ hybridization (FISH) and karyotyping then followed by array comparative genomic hybridization (aCGH). Of the 386 cases retrieved from the past decade, common aneuploidies were detected in 137 cases (35.5%), other chromosomal structural rearrangements were detected in four cases (1%), and pCNV were detected in five cases (1.3%). The relative frequencies for common aneuploidies suggested an under detection of sex chromosome aneuploidies. Approximately 9.5% of cases with common aneuploidies showed a mosaic pattern. Inconsistent results between FISH and karyotyping were noted in cases with pseudo-mosaicism introduced by culture artifact or variable cellular proliferation from cells with mosaic karyotypic complements under in vitro cell culture. Based on findings from this case series, cell-based FISH and karyotyping should be performed to detect common aneuploidies, structural chromosomal abnormalities, and mosaic pattern. DNA-based aCGH and reflex FISH should be performed to detect and confirm genomic imbalances and pCNV. Practice points to ensure the diagnostic accuracy and efficacy were summarized.

## 1. Introduction

Major chromosomal abnormalities have been detected in 0.65% of newborns from combined surveys during 1969–1982; and approximately 75% of these chromosomal abnormalities are common aneuploidies involving chromosomes 13, 18, 21, X, and Y [1]. Due to the severe phenotypes from these common aneuploidies, continuous efforts have been made to improve the accuracy and efficacy for detecting these abnormalities in prenatal diagnosis. Since the 1980s, prenatal diagnosis of pregnancies at risk of common aneuploidies have been indicated by abnormal maternal serum screening, advanced maternal age, abnormal ultrasound findings on the fetus, and family history of chromosome abnormalities. Routine chromosome karyotyping, fluorescent in situ hybridization (FISH), multiplex ligation-dependent probe amplification (MLPA), and array comparative genomic hybridization (aCGH) analyses have been implemented using invasive chorionic villus sampling (CVS) and amniotic fluid (AF) specimens [1,2,3,4,5]. In the past decade, changes in and efficacies of indications for invasive prenatal diagnosis have been noted, especially with the introduction of a highly effective screening of common aneuploidies by non-invasive prenatal testing (NIPT) using next-generation sequencing on maternal serum cell free fetal DNA [6,7,8]. Despite all these technical advancements, correlating cell-based FISH and chromosome results to resolve pseudo-mosaicism by culture artifact, fetoplacental discrepancy by confined placental mosaicism (CPM), and true fetal mosaicism (TFM) is still a major challenge in both laboratory operation and result interpretation [9,10,11]. To evaluate the diagnostic outcome and technical limitations of cell-based FISH and chromosome analyses and DNA-based aCGH testing on detecting common aneuploidies and other cytogenomic abnormalities, a retrospective analysis was performed on a case series retrieved from the 2008–2017 period. The results provided important practice recommendations for an integrated cell-based and DNA-based approach to ensure the accuracy and efficacy on detecting common aneuploidies and other cytogenomic abnormalities in the current prenatal setting.

## 2. Materials and Methods 

Yale clinical cytogenetics laboratory performed chromosome, FISH, and aCGH tests on CVS and AF specimen from prenatal clinics. Aneuvysion FISH was performed using Food and Drug Administration (FDA) approved probes consisting of three α-satellite DNA centromeric probes of D18Z1 at 18cen, DXZ1 at Xcen and DYZ3 at Ycen and two locus-specific probes of the *RB1* gene at 13q14 and the D21S259 locus at 21q22 (Abbott/Vysis. Downers Grove, IL, USA). Reflex FISH was performed using targeted locus-specific probes (Cytocell Inc. Cambridge, UK). The aCGH was performed using Agilent Human Genome CGH microarray 180 K kit (Agilent Technologies, Inc., Santa Clara, CA, USA) following manufacturer’s instruction and a standardized protocol validated by the laboratory [12]. The detected genomic imbalances were designated per the GRCh37/hg19 assembly in the University of California Santa Cruz (UCSC) Human Genome browser (http://genome.ucsc.edu/).

To evaluate the diagnostic yield and abnormal findings, prenatal cases analyzed first by combined aneuvysion FISH and karyotyping assays and then followed up aCGH during the 2008–2017 period were retrieved from the laboratory’s CytoAccess database [13]. There were 386 prenatal cases analyzed by aneuvysion FISH and karyotyping, and 162 cases of them with a normal karyotype or a structural chromosome abnormality were further analyzed by aCGH. Overall, a trend of a significant reduction of invasive prenatal procedures, a relative stable utility of aneuvysion FISH and chromosome analyses, and a steady increase of aCGH analysis in prenatal cases was noted in the laboratory’s prenatal operation. The diagnostic yield was presented as the abnormality detection rate (ADR) by dividing number of abnormal cases by the total number of cases. Relative frequencies of common aneuploidies were given by dividing the number of abnormal cases of a specific chromosome by the total number of cases with common aneuploidies. Abnormal mosaic cases were evaluated on a case-by-case basis to resolve technical discrepancy and to interpret its clinical implication. Other abnormal findings including the chromosomal structural rearrangements and pathogenic copy number variants (pCNV) detected by aCGH were also summarized.

For this retrospective study, there were no pre-study requirements on the patient’s specimens and clinical indications and there were no post-study interaction and intervention with the patients. This project was categorized as a chart review retrospective study and deemed exempted from Institutional Review Boards (IRB) approval and waiver of consent based on the policy of Yale University IRB.

## 3. Cytogenomic Results

### 3.1. Clinical Indications and Common Aneuploidies Detected

**Figure 1:**
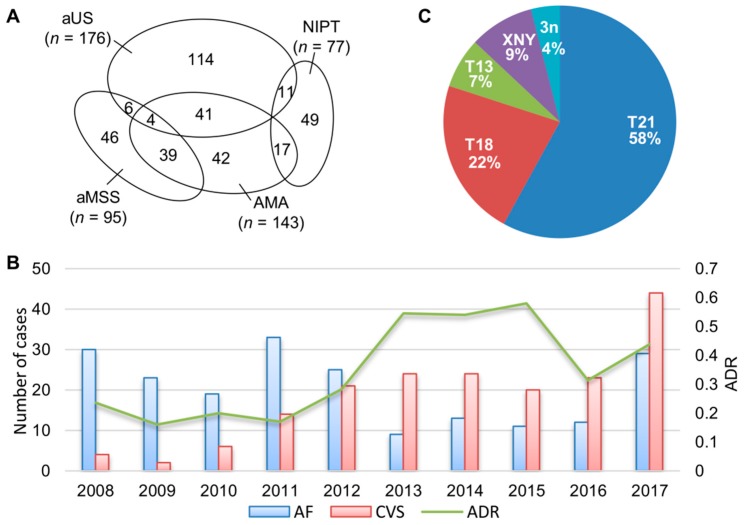
Laboratory annual caseload and common aneuploidies detected. (**A**) The number of prenatal cases with single and overlapping clinical indications of abnormal ultrasound findings (aUS), advanced maternal age (AMA), abnormal maternal serum screening (aMSS), and non-invasive prenatal testing (NIPT). (**B**) Annual caseload and abnormality detection rate (ADR) for common aneuploidies from aneuvysion fluorescence in situ hybridization (FISH) and chromosome analyses on amniotic fluid (AF) and chorionic villus sampling (CVS) cases (**C**) A pie chart shows relative frequencies for trisomy 21 (T21), trisomy 18 (T18), trisomy 13 (T13), sex chromosome aneuploidies (XNY/X), and triploidy (3n).

Combined aneuvysion FISH and chromosome analyses were performed on a total of 386 prenatal cases; annual caseload for AF and CVS ranged from 9 to 33 cases and 2 to 44 cases, respectively. Clinical indications for these cases included abnormal ultrasound anomalies (aUS, *n* = 176), advanced maternal age (AMA, *n* = 143), abnormal maternal serum screening (aMSS *n* = 95), NIPT (*n* = 77), and familial history of chromosome abnormalities (*n* = 17). The number of cases referred by single or overlapping clinical indications of aUS, AMA, aMSS, and NIPT is shown in Figure 1. 

Common aneuploidies were detected in 137 cases; and the diagnostic yield measured by ADR for common aneuploidies was 35.5% (137/386). The relative frequencies of trisomy 21, trisomy 18, trisomy 13, sex chromosome aneuploidies (45,X or 47,XXX/XYY/XXY), and triploidy were calculated as 58% (80/137), 22% (30/137), 7% (9/137), 9% (12/137), and 4% (6/137), respectively. Of all these cases with common aneuploidies, two cases were caused by a Robertsonian translocation: one with a trisomy 13 by 46,XY + 13,rob(13;13)(q10;q10), and another with a trisomy 21 of maternal origin by 46,XY,rob(14;21)(q10;q10)mat,+ 21. Robertsonian translocations accounted for about 1.5% (2/137) of total common aneuploidies. Mosaic patterns involving chromosomes 21, 18, 13 and X were detected in 13 cases. The annual caseload, ADR, and relative frequencies for common aneuploidies are shown in Figure 1. The average diagnostic yield for the 2008–2012 and 2013–2017 period was 21% and 48%, respectively. Non-invasive prenatal testing has been applied to prenatal cases since 2013. In this case series, NIPT was used in 37% of prenatal cases (77 out of the 206 cases) in the 2013–2017 interval. Of the 63 cases with abnormal results from NIPT, 84% (53/63) were confirmed by the FISH and karyotyping (Table 1). Discording NIPT and cytogenetic results were noted in one case and two twin pregnancies suspected for trisomy 21, four cases suspected for 45,X, and one case for 47,XXY by NIPT. 

The high positive predicative value from NIPT contributed to the significant improvement of diagnostic yield in the 2013–2017 interval.

### 3.2. Cases with Mosaic Patterns

The 13 cases with a mosaic pattern accounted for 9.5% of total cases with common aneuploidies. The mosaic findings including five cases for chromosome 21, two cases for chromosome 18, one case for chromosome 13, and five cases for monosomy X are listed in Table 2.

Consistent aneuvysion FISH and chromosome results were noted in seven cases, while inconsistent results were noted in six cases. In case 3, aneuvysion FISH showed trisomy 21 in 96% of directly prepared uncultured villus cells but chromosome analysis showed a mosaic pattern with cells carrying trisomy 21, monosomy 21, and disomy 21. A reflex FISH test using locus-specific probes for the *ETV6* gene at 12p13 and the *RUNX1* gene at 21q22 confirmed the trisomy 21 and monosomy 21 pattern in cultured villus cells. Since monosomy 21 was incompatible with life and caused early embryonic lethal and miscarriage in vivo, the emerging cells with monosomy 21 were most likely from mitotic non-disjunction under in vitro cell culture. In case 4, aneuvysion FISH showed a trisomy 21 in all directed prepared villus cells but karyotype showed a mosaic pattern of trisomy 21 and trisomy 5 in cultured villus cells. Since disomic 21 was not seen by FISH, the cells with trisomy 5 in one culture were most likely a culture artifact and the mosaic pattern was considered as a pseudo-mosaicism. In case 6, aneuvysion FISH showed a normal male pattern on directly prepared villus cells and karyotyping detected a true mosaic pattern with an isochromosome of 18q in cultured villus cells. FISH using the centromeric probe D18Z1 would not detect the duplication of 18q and deletion of 18p in this isochromosome of 18q. In case 7, consistent aneuvysion FISH and karyotype results for a mosaic pattern of trisomy 18 were noted on CVS. Follow up amniocentesis enabled the detection of a mosaic pattern of trisomy 18 in 18% of directly prepared uncultured amniocytes by aneuvysion FISH but a normal male karyotype from the analysis of 20 colonies of cultured amniocytes. A reflex FISH using locus-specific probes for the *IGH* gene at 14q32 and the *BCL2* gene at 18q21 on one in situ cultured slide noted a normal pattern of two signals for each probe. Chromosome analysis of 20 metaphases could rule out a mosaic pattern in more than 14% of cells with a 95% confidence level [14]; the reflex FISH analysis on cultured amniocytes from one slide confirmed the chromosome finding but would not rule out low level mosaicism by the limited number of cells or colonies analyzed. The discrepancy between chromosome and FISH results could be caused by the low percentage of abnormal cells with trisomy 18 or the low mitotic index of these cells under in vitro cell culture. In case 11, aneuvysion FISH result showed monosomy X, disomy X, and trisomy X in 8%, 69%, and 23% of directly prepared villus cells, respectively; karyotype detected a mosaic pattern of monosomy X and trisomy X. The discrepancy between chromosome and FISH results was likely due to the low mitotic index of cells with disomy X. In case 13, aneuvysion FISH detected monosomy X in 62% of directly prepared villus cells but karyotype showed a normal 46, XX female pattern. Further aCGH analysis using DNA extracted from villus cells confirmed the mosaic pattern of monosomy X. The discrepancy between chromosome and FISH results was likely due to the low mitotic index of cells with monosomy X. The last two cases demonstrated that low mitotic activity under in vitro cell culture might occur in cells with a normal karyotype or an abnormal karyotype.

### 3.3. Chromosomal Structural Abnormalities and Pathogenic Copy Number Variants

In additional to common aneuploidies, structural chromosome abnormalities were detected in four cases by karyotyping and pCNV were detected in five cases by aCGH (Table 3).

Parental chromosome analysis was always recommended and performed for prenatal cases with a structural chromosome abnormality. Apparently balanced translocations were noted in case 14 with a paternal t(4;19)(q25;q13.3), in case 15 with a de novo t(6;17) (p21.1;q24), and in case 16 with a de novo t(9;21)(p23;q21). Case 17 had a derivative chromosome 5 from a maternal t(5;7)(p15.3;q21.1) and therefore showed a segmental deletion of 5p15.3-pter and duplication of 7q21.1-qter.

Case 18 was a male fetus with a 6.532 Mb duplication at 7p12.1–p11.2 (51,277,556–57,809,908) detected by aCGH, and this interstitial duplication was confirmed by karyotyping on cultured villus cells. This duplication includes the *COBL, POM121L12, HPVC1, VSTM2A, SEC61G, EGFR, VOPP1, FKBP9L, U6, CO9, SEPT14, ZNF713, MRPS17, GBAS, PSPH, CCT6A, SUMF2, PHKG1, CHCHD2, NUPR1L, ZNF479, TRNA,* and *ZNF716* genes. Among genes within this duplication, three have entries in the Online Mendelian Inheritance in Man (OMIM, https://www.omim.org/). Mutations in the *EGFR* gene cause autosomal recessive neonatal inflammatory skin and bowel disease-2 (NISBD2, OMIM#616069) and lung cancer (OMIM#211980). Mutations in the *PSPH* gene cause autosomal recessive phosphoserine phosphatase deficiency (PSPHD, OMIM#614023). Heterozygous mutation in the *CHCHD2* gene may be a rare cause of autosomal dominant Parkinson disease 22 (PARK22, OMIM#616710). Maternally inherited duplication, dup(7) (p11.2p12), was reported in a family with three carriers showing mild cognitive deficit [15]. The duplication at 7p12.1–p11.2 observed in this fetus was a likely pCNV. Follow up aCGH analysis on both parents revealed a paternal origin of this pCNV. The father had a history of hypertrophic cardiomyopathy and his brother died suddenly at age 26. The fetus was noted to have increased nuchal translucency by prenatal ultrasound scan and was born at term. Physical examination at three-months of age appeared well-developed except for mild plagiocephaly. His echocardiogram showed small mild muscular atrial and ventricular septal defects.

Case 19 showed a normal female karyotype by chromosome analysis on cultured amniocytes but aCGH detected a 2.488 Mb deletion at 17p13.3–p13.2 (1,078,112–3,566,410). This deletion includes the *YWHAE* and *PAFAH1B1* genes. A reflex FISH analysis confirmed the deletion of *PAFAH1B1* gene. Deletions at 17p13.3 cause autosomal dominant Miller-Dieker Lissencephaly syndrome (MDLS, OMIM#247200). Deletions involving the *YWHAE* gene showed significant growth restriction, cognitive impairment, and shared craniofacial features, including tall vertex, prominent forehead, broad nasal root, and epicanthal folds; deletions involving the *PAFAH1B1* gene presented with seizures, significant developmental delay, and classic lissencephaly [16]. Postnatal FISH test confirmed the deletion at 17p13.3–p13.2. Clinical evaluation showed infantile spasms, seizures, and lissencephaly. The baby died at one-year of age.

Case 20 was detected with a derivative chromosome 22 from a paternal t(16;22) (p12.2;q13.31) by chromosome analysis on cultured villus cells; aCGH defined a 22.549 Mb duplication of 16p13.3–p12.2 and a 6.688 Mb deletion of 22q13.31–q13.33. A reflex FISH analysis detected a deletion involving the *SHANK3* gene at 22q13.3. This terminal 22q13.3 deletion causes Phelan–McDermid syndrome which is characterized by neonatal hypotonia, global developmental delay, normal to accelerated growth, absent to severely delayed speech, autistic behavior, and minor dysmorphic features (OMIM#606232). The parents decided to terminate this pregnancy.

Case 21 had a normal male karyotype by chromosome analysis on cultured amniocytes and a 2.131 Mb deletion at 22q11.21 (18,894,835–21,025,713) detected by aCGH. A reflex FISH test was performed using dual color probes for the *TBX1* gene at 22q11.21 and the *SHANK3* gene at 22q13.3. An abnormal pattern with a loss of the *TBX1* signal in one chromosome 22 was noted in all 25 metaphases examined. Recurrent deletions at 22q11.21 cause DiGeorge syndrome (OMIM#188400). The baby was delivered at 35 weeks and found micrognathia, ASD, VSD, esophageal atresia, and tracheoesophageal fistula at birth. He had a prolonged stay at newborn intensive care unit (NICU) with a surgery repair of ASD, VSD, and tracheoesophageal fistula. A follow up echocardiogram at 3-months of age showed an excellent repair with no atrial level shunt, a small residual, pressure and volume restrictive VSD, and adequate biventricular function.

Case 22 had a normal female karyotype by chromosome analysis on cultured amniocytes and a 2.834 Mb duplication at 22q11.21-q11.23 (21,808,950–24,643,108) detected by aCGH. A reflex FISH test was performed using dual color probes for the *ABL1* gene at 9q34.1 and the *BCR* gene at 22q11.21. Of the 200 interphase cells examined, 100% showed two signals for the *ABL1* probe and three signals (one independent and two twin-spot like) for the *BCR* probe. This result confirmed the 22q11.21-q11.23 duplication. Duplications of 22q11.21-q11.23 distal to the 22q11.21 microdeletion syndrome region have been identified in familial and de novo cases; the patients showed variable development delay with no speech or walking, and other neurological features vary from normal to profound hypotonia and/or severe seizures and some contradicting features like macrocephaly and microcephaly [17]. The fetus was noted to have a mildly hypoplastic right ventricle by prenatal ultrasound scan. She was born at term and remained in the NICU for eight days with required intermittent O_2_. A follow up echocardiogram at 7-months of age showed resolved bilateral peripheral pulmonary artery stenosis and small ASD with mostly left to right flow.

## 4. Discussion

From these 386 prenatal cases analyzed by aneuvysion FISH and karyotyping, 137 cases were detected with common aneuploidies. Of which, 89% were complete aneuploidies, 1.5% were derived from a Robertsonian translocation, and 9.5% showed a mosaic pattern. The relative frequencies of common aneuploidies showed 58% for trisomy 21, 22% for trisomy 18, 7% for trisomy 13, and 9% for sex chromosome aneuploidies. The occurrence of chromosomal abnormalities in liveborn babies were 0.12% for trisomy 21, 0.013% for trisomy 18, 0.004% for trisomy 13, and 0.34% for sex chromosome aneuploidies [1]. A comparison between prenatal and liveborn findings suggested an over-representation of trisomy 18 and trisomy 13 and under-representation of sex chromosome aneuploidies in the current prenatal setting. Most fetuses with trisomy 18 or 13 were aborted in the first trimester but most sex chromosome aneuploidies except monosomy X could survive to term and grow to adult. Current prenatal practice is effective on detecting autosomal aneuploidies but may be suboptimal for detecting sex chromosome aneuploidies. A NIPT screening using circulating fetal cells from maternal serum would likely to improve the efficacy on detecting sex chromosome aneuploidies [6].

The analysis and the interpretation of a mosaic pattern should be cautious in the prenatal diagnosis. As shown in Table 1, pseudo-mosaicism could be introduced by in vitro cell culture (case 4) and inconsistent results between aneuvysion FISH and karyotype could result from variable proliferation of cells with different karyotype under in vitro cell culture (cases 3, 11 and 13). Mosaic patterns observed on CVS could be confined to the placenta, follow up amniocentesis were always recommended [9,10,11]. Of the ten mosaic patterns detected on CVS, only one (case 7) with a mosaic pattern of trisomy 18 was followed by an amniocentesis. A mosaic pattern of trisomy 18 was seen in 18% of uncultured amniocytes and a normal male karyotype was detected by chromosome analysis of 20 colonies. This case demonstrated the technical limitations on resolving discrepant chromosome and aneuvysion FISH results and the challenge on reporting CPM or a low-level mosaicism. To resolve pseudo-mosaicism and variant mosaic patterns introduced by in vitro cell culture, a reflex FISH using targeted locus-specific probes to confirm a mosaic pattern on directly prepared uncultured amniocytes or villus cells is highly recommended [18].

Structural chromosomal rearrangements were noted in two cases with a Robertsonian translocation and in one case with a mosaic pattern of an isochromosome of 18q. Other chromosome structural rearrangements undetectable by aneuvysion FISH were noted in four cases (cases 14–17) by chromosome analysis. These results support that chromosome analysis is still an important method on detecting balanced and unbalanced structural rearrangements [19]. Furthermore, pCNV were detected in five cases (cases 18–22) by aCGH analysis. Since aCGH was performed on 60% (162/269) of prenatal cases, it was estimated that aCGH performed on more cases with normal chromosome findings could increase the diagnostic yield. This observation and our previous experience proved that aCGH can further define the genomic imbalances in structural chromosomal abnormalities and improve the diagnostic yield in prenatal diagnosis [20,21,22]. To confirm pCNV detected by aCGH, a reflex FISH using targeted locus-specific probes should be performed. The clinical significance of pCNV should be interpreted in an evidence-based manner to facilitate genetic counseling and clinical assessment by ultrasonography [23].

**Figure 2:**
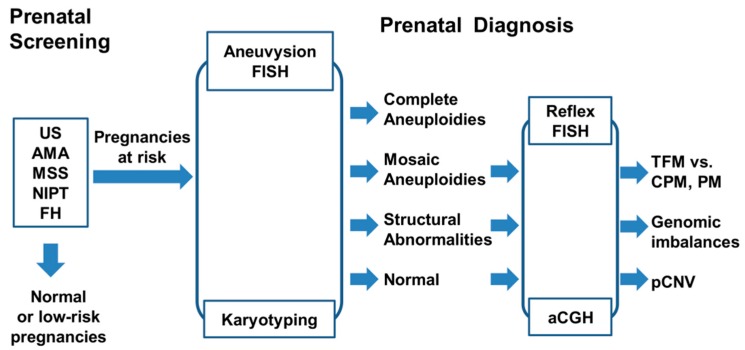
A workflow of prenatal screening and diagnosis of common aneuploidies and other cytogenomic abnormalities. Results from non-invasive prenatal testing (NIPT), maternal serum screening (MSS), advanced maternal age (AMA), ultrasound examination (US), and family history (FH) of chromosomal abnormalities have been used as indications to screen for pregnancies at risk. Integrated aneuvysion FISH, karyotyping, array comparative genomic hybridization (aCGH), and reflex FISH are performed to detect complete aneuploidies, mosaic aneuploidies (TFM, true fetal mosaicism versus CPM, confined placenta mosaicism and PM, pseudo-mosaicism), structural abnormalities, and pathogenic copy number variants (pCNV).

The implementation of next generation sequencing for non-invasive prenatal screening of common aneuploidies on maternal serum cell free fetal DNA has resulted in a significant reduction in invasive prenatal procedures and significantly increased diagnostic yield in prenatal diagnosis [7,8]. A workflow for multi-indicator prenatal screening of pregnancies at risk and cytogenetic diagnosis of common aneuploidies and other cytogenomic abnormalities is shown in Figure 2.

In a clinical cytogenetics laboratory, several practice points for improving diagnostic accuracy and efficacy are outlined as the following:

- Aneuvysion FISH provides a quick and reliable testing of common aneuploidies. Once a mosaic pattern is detected, a reflex FISH test using targeted locus-specific probes should be considered to confirm the mosaic pattern in directly prepared cells and to verify the inconsistent findings from cultured cells. The cut-off value and frequencies of false positive and false negative from FISH tests should be monitored carefully [24,25]. Abnormal results detected by a quick FISH test should be interpreted with cautions and communicated clearly with genetic counselors and physicians. It is important to have a disclaimer clearly state that false positive or negative results as well as maternal cell contamination have been demonstrated in prenatal FISH analysis; the American College of Medical Genetics recommends that irreversible therapeutic action should not be initiated on the basis of FISH results alone.
- Karyotyping is still a valuable method on detecting common aneuploidies, balanced or unbalanced structural rearrangements, and mosaic patterns. Follow up amniocentesis should always be recommended on mosaic findings from CVS. However, the low analytical resolution by G-banding and the limitation on resolving low-level mosaicism should be taken into consideration.
- Efforts should be applied to resolve pseudo-mosaicism, CPM, and TFM in prenatal diagnosis [9,10,11]. Technically, additional workup to define the level I, II, and III of mosaicism should be performed according to the laboratory protocol and documented in the worksheet. The distinction of pseudo-mosaicism, CPM, and TFM should be based on completed chromosome and FISH results. Pseudo-mosaicism by culture artifact and variant mosaic patterns introduced by in vitro cell culture should be resolved by a reflex FISH preferably in uncultured cells. Technical limitations in analyzing low level mosaic patterns should be described clearly [14]. Complete chromosome and aneuvysion FISH results and adequate interpretation should be provided in a timely fashion.
- Reporting of mosaic patterns should be based on laboratory findings and current evidence. Current survey indicated that CPM and TFM accounted for 86% and 14% of prenatal mosaic cases, respectively. It was estimated that the risk of finding a mosaicism in CVS after a high-risk cell free DNA test result for trisomy 21, trisomy 18, trisomy 13, and monosomy X is 2%, 4%, 22%, and 59%, respectively. After the detection of a mosaic pattern in CVS, the likelihood of fetal confirmation for trisomy 21, trisomy 18, trisomy 13 and monosomy X is 44%, 14%, 4%, and 26%, respectively [11]. A web-based database for chromosome mosaicism (http://mosaicism.bcchr.ca/) could also be helpful in interpreting mosaic findings. Prenatal mosaic findings should be reported and communicated directly to genetic counselors and referring physicians.
- The aCGH should be performed for cases with abnormal structural chromosomal rearrangements and normal chromosome findings. Any pCNV detected by aCGH should be confirmed by a reflex FISH using targeted locus-specific probes. The clinical significance of pCNV should be interpreted based on evidences from current clinical database and literature. Follow up parental study should be recommended for cases with structural chromosome rearrangements and pCNV.

## 5. Conclusions

A clinical cytogenetics laboratory performing prenatal diagnosis should understand the advantages and disadvantages of cell-based FISH and chromosome analyses and DNA-based aCGH analysis. An integrated approach following the proposed practice points could improve the diagnostic accuracy and efficiency on detecting common aneuploidies and other cytogenomic abnormalities.

## Figures and Tables

**Table 1:** The abnormal NIPT cases confirmed by FISH and karyotyping.

	Trisomy 21	Trisomy 18	Trisomy 13	45,X	47,XXX	47,XXY	47,XYY	69,XXX	Total
NIPT	38 *	11	3	7	1	1	1	1	**63**
FISH	33	11	3	3 **	1	0	1	1	**53**
Karyotyping	33	11	3	2	1	0	1	1	**52**

* including two pairs of twins, ** one mosaic case (#13 in Table 2) detected by FISH but not by karyotyping; NIPT, non-invasive prenatal testing; FISH, fluorescent in situ hybridization.

**Table 2:** Chromosome and FISH results on mosaic cases of common aneuploidies.

Case No.	Sample	Karyotype Results	Aneuvysion FISH Results
**1**	AF	mos 47,XY,+21[15]/46,XY[2]	nuc ish(DXZ1x1,DYZ3x1,D18Z1x2)[40] nuc ish(RB1x2,D21S259x3)[41/50]/(RB1,D21S259)x2[9/50]
**2**	CV	mos 47,XX,+21[12]/46,XX[3]	nuc ish(DXZ1,D18Z1)x2[100] nuc ish(RB1x2,D21S259x3)[75/100]/(RB1,D21S259)x2[25/100]
**3**	CV	mos 47,XY,+21[2]/45,XY,-21[12]/46,XY[1] nuc ish(ETV6x2,RUNX1x3)[56/200]/(ETV6x2,RUNX1x1)[113/200]	nuc ish(DXZ1x1,DYZ3x1,D18Z1x2)[100] nuc ish(RB1x2,D21S259x3)[96/100]/(RB1,D21S259)x2[4/100]
**4**	CV	mos 47,XX,+21[17]/47,XX,+5[3]	nuc ish(DXZ1,D18Z1)x2[30], nuc ish(RB1x2,D21S259x3)[30]
**5**	AF	mos 47,XX,+21[4]/46,XX[11]	nuc ish(RB1,D21S259)x2[195/200]/(RB1x2,D21S259x3)[5/200]
**6**	CV	mos 46,XY,i(18)(q10)[7]/46,XY[8]	nuc ish(DXZ1x1,DYZ3x1,D18Z1x2)[50] nuc ish(RB1,D21S259)x2[100]
**7**	CV	mos 47,XY,+18[2]/46,XY[48]	nuc ish(DXZ1x1,DYZ3x1,D18Z1x2)[88/100]/(DXZ1x1,DYZ3x1,D18Z1x3)[12/100] nuc ish(RB1x2,D21S259)x2[30]
	AF	46,XY.nuc ish(IGH,BCL2)x2[200]	nuc ish(DXZ1x1,DYZ3x1,D18Z1x2)[49/60]/(DXZ1x1,DYZ3x1,D18Z1x3)[11/60]
**8**	CV	mos 47,XX,+13[6]/46,XX[14]	nuc ish(RB1x3,D21S259x2)[46/50]/(RB1,D21S259)x2[4/50]
**9**	CV	mos 45,X[5]/46,XX[15]	nuc ish(DXZ1x2)[252/300]/(DXZ1x1)[48/300]
**10**	CV	mos 45,X[6]/46,XX[14]	nuc ish(DXZ1x1,D18Z1x2)[7/100]/(DXZ1,D18Z1)x2[93/100] nuc ish(RB1,D21S259)x2[100]
**11**	CV	mos 45,X[4]/47,XXX[16]	nuc ish(DXZ1,D18Z1)x2[8/100]/(DXZ1x1,D18Z1x2)[69/100]/(DXZ1x3,D18Z1x2)[23/100] nuc ish(RB1,D21S259)x2[75]
**12**	AF	mos 45,X[20]/47,XXX[1]	nuc ish(DXZ1x1,D18Z1x2)[16/25]/(DXZ1x3,D18Z1x2)[9/25] nuc ish(RB1,D21S259)x2[25]
**13**	CV	46,XX	nuc ish(DXZ1x2,D18Z1x2)[38/100]/(DXZ1x1,D18Z1x2)[62/100]

**Table 3:** Structural chromosomal abnormalities and pathogenic copy number variants.

Case No.	Sample	Chromosome Results	Reflex Locus-Specific FISH Results	aCGH Results (hg19)
**14**	AF	46,XX,t(4;19)(q25;q13.3)pat		
**15**	AF	46,XY,t(6;17)(p21.1;q24)dn		
**16**	CV	46,XX,t(9;21)(p23;q21)dn		
**17**	AF	46,XX,der(5)t(5;7)(p15.3;q21.1)mat		
**18**	CV	46,XY,dup(7)(p11.2p12.1)pat		arr 7p12.1p11.2(51,277,556-57,809,908)x3pat
**19**	AF	46,XX	nuc ish(PAFAH1B1x1,RAI1x2)[25]	arr 17p13.3p13.2(1,078,112-3,566,410)x1
**20**	CV	46,XY,der(22)t(16;22)(p12.2;q13.31)pat	ish der(22)t(16;22)(TBX1+,SHANK3-)[25]	arr 16p13.3p12.2(96,766-22,645,765)x3, 22q13.31q13.33(44,505,356-51,193,680)x1
**21**	AF	46,XY	ish del(22)(q11.21q11.21)(TBX1-,SHANK3+)[25]	arr 22q11.21(18,894,835-21,025,713)x1
**22**	AF	46,XX	nuc ish(ABL1x2,BCRx3)[200]	arr 22q11.21q11.23(21,808,950-24,643,108)x3dn
